# Dynamics of *Streptococcus mutans* Transcriptome in Response to Starch and Sucrose during Biofilm Development

**DOI:** 10.1371/journal.pone.0013478

**Published:** 2010-10-19

**Authors:** Marlise I. Klein, Lena DeBaz, Senyo Agidi, Herbert Lee, Gary Xie, Amy H.-M. Lin, Bruce R. Hamaker, José A. Lemos, Hyun Koo

**Affiliations:** 1 Center for Oral Biology and Eastman Department of Dentistry, University of Rochester Medical Center, Rochester, New York, United States of America; 2 Department of Microbiology and Immunology, University of Rochester Medical Center, Rochester, New York, United States of America; 3 Los Alamos National Laboratory, Los Alamos, New Mexico, United States of America; 4 Department of Food Science, Whistler Center for Carbohydrate Research, Purdue University, West Lafayette, Indiana, United States of America; National Institute of Allergy and Infectious Diseases, National Institutes of Health, United States of America

## Abstract

The combination of sucrose and starch in the presence of surface-adsorbed salivary α-amylase and bacterial glucosyltransferases increase the formation of a structurally and metabolically distinctive biofilm by *Streptococcus mutans*. This host-pathogen-diet interaction may modulate the formation of pathogenic biofilms related to dental caries disease. We conducted a comprehensive study to further investigate the influence of the dietary carbohydrates on *S. mutans*-transcriptome at distinct stages of biofilm development using whole genomic profiling with a new computational tool (MDV) for data mining. *S. mutans* UA159 biofilms were formed on amylase-active saliva coated hydroxyapatite discs in the presence of various concentrations of sucrose alone (ranging from 0.25 to 5% w/v) or in combination with starch (0.5 to 1% w/v). Overall, the presence of sucrose and starch (suc+st) influenced the dynamics of *S. mutans* transcriptome (vs. sucrose alone), which may be associated with gradual digestion of starch by surface-adsorbed amylase. At 21 h of biofilm formation, most of the differentially expressed genes were related to sugar metabolism, such as upregulation of genes involved in maltose/maltotriose uptake and glycogen synthesis. In addition, the *groEL/groES* chaperones were induced in the suc+st-biofilm, indicating that presence of starch hydrolysates may cause environmental stress. In contrast, at 30 h of biofilm development, multiple genes associated with sugar uptake/transport (e.g. maltose), two-component systems, fermentation/glycolysis and iron transport were differentially expressed in suc+st-biofilms (vs. sucrose-biofilms). Interestingly, *lytT* (bacteria autolysis) was upregulated, which was correlated with presence of extracellular DNA in the matrix of suc+st-biofilms. Specific genes related to carbohydrate uptake and glycogen metabolism were detected in suc+st-biofilms in more than one time point, indicating an association between presence of starch hydrolysates and intracellular polysaccharide storage. Our data show complex remodeling of *S. mutans*-transcriptome in response to changing environmental conditions *in situ*, which could modulate the dynamics of biofilm development and pathogenicity.

## Introduction

Dental caries continues to be the single most common biofilm-dependent oral infectious disease worldwide [1]. Dental caries result from the interaction of specific bacterial and salivary constituents with dietary carbohydrates in biofilms tightly adherent on the tooth surface [1], [2]. *Streptococcus mutans*, a member of the oral microbial community, plays a key role in modulating the transition from non-pathogenic form to highly cariogenic biofilms [3], although additional organisms may be also associated with this ubiquitous disease (as reviewed in [4]). This bacterium is able to thrive and compete in the complex biofilm microbiome, and contribute to the pathogenesis of dental caries because it: (i) effectively utilizes dietary sucrose to rapidly synthesize exopolysaccharides (EPS) through glucosyltransferases and a fructosyltransferase that adsorb to surfaces, (ii) adheres tenaciously to glucan-coated surfaces, and (iii) is highly acidogenic and aciduric [2], [5].

Sucrose and starch, the main dietary carbohydrates in modern societies, are potentially more cariogenic in combination than is either alone, both in animals and humans [6]–[8]. Host salivary α-amylases digest starches to maltose, maltodextrins and other oligosaccharides and some of these starch hydrolysates can be incorporated during glucan synthesis by glucosyltransferases (Gtfs) via acceptor reactions [9], [10]. More importantly, these host- and bacterial-derived enzymes adsorb to the pellicle in active form [10]. In the presence of sucrose and starch, surface adsorbed-GtfB and -amylase acting in concert increase the synthesis of structurally distinct glucans, which provide enhanced bacterial binding (including *S. mutans*) to apatitic surfaces [10]. *S. mutans* has multiple sugar transport systems involved in the uptake of starch hydrolysates (e.g. maltose and maltotriose) [11]–[13], which can be further metabolized into acids [14].

Furthermore, we have collected evidence that the interaction of sucrose and starch through surface-adsorbed salivary α-amylase and Gtf enzymes (particularly GtfB) modulates *in situ* the development of biofilms that are both structurally and metabolically distinctive [15], [16]. These interactions promote formation of biofilms with elevated amounts of EPS and increased acidogenicity [15] by up-regulating the expression of *gtfB* and *msm* operon genes [15], [16]. The *gtfB* gene (and its GtfB product) is a recognized virulence factor associated with the pathogenesis of dental caries in rodents and in humans [17], [18]. Therefore, analysis of the dynamics of transcriptomic responses of *S. mutans* to starch and sucrose during the biofilm formation process would enhance our understanding of the molecular mechanisms involved with the biochemical and structural changes, and increased pathogenicity observed previously [6]–[8], [15], [16].

In this study, we used a whole genomic profiling approach to further characterize how *S. mutans* responds to this unique host-pathogen-diet interaction at distinct time points over the course of biofilm formation on a saliva-coated hydroxyapatite surface. In addition, we developed a new software to analyze the microarray data, focusing on how specific transcriptome changes may be associated with enhanced biofilms accumulation, survival and virulence of this pathogen.

## Materials and Methods

### Biofilm preparation

Hydroxyapatite discs (2.93 cm^2^, Clarkson Chromatography Products, Inc., South Williamsport, PA) were coated with amylase-active, filter-sterilized clarified human whole saliva (sHA) [15], [16]. Our previous studies demonstrated that salivary amylase adsorbed on the HA surface is most active during the first 48 h after pellicle formation, digesting about 50% of starch available in the culture medium [15], [16]. Biofilms of *S. mutans* UA159 (ATCC 700610) were formed on sHA discs placed in a vertical position in batch cultures at 37°C in 5% CO_2_, as described elsewhere [19]. The biofilms were grown in ultrafiltered (10-kDa-cutoff membrane; Prep/Scale, Millipore, MA) buffered tryptone yeast-extract broth (UFTYE) at pH 7.0 [20], containing specific concentrations of sucrose and/or starch (soluble starch −80% amylopectin and 20% amylose; Sigma Chemical Company, St Louis, MO). The concentrations of 0.5% sucrose, 0.5% sucrose +1% starch and 1% sucrose were selected based on biochemical and molecular analyses from our preliminary studies (see data in Appendix S1) and previously published data [15], [16], [21]. The biofilms were grown in batch cultures at 37°C in the presence of 5% CO_2_ for 44 h. During the first 20 h, the organism was grown undisturbed to allow initial biofilm formation. The culture media was replaced at 20 h and 30 h of biofilm formation. Biofilms were analyzed by RT-qPCR at 21, 24, 30, 31 and 34 h, and by biochemical assays at 30 and 44 h.

### RNA isolation and RT-qPCR

RNA was extracted and purified from biofilms at distinct stages of microcolonies development (21, 24, 30, 31, and 34 h) using standard protocols [22]. The developmental stages of *S. mutans* biofilms were characterized previously [21], [23], which varies from initial microcolonies assembly across the apatite surface (at 20 h) to vertical growth and merging process followed by further increase in size and thickness (from 20 to 30 h and beyond). All purified RNAs presented RNA integrity number (RIN) ≥8.5 (Agilent 2100 electrophoresis bioanalyzer, Agilent Technologies, Santa Clara, CA, USA). The reverse transcriptase PCR, and quantitative amplification conditions were similar to those described previously [20]. The primers were designed using Beacon Designer 2.0 software (Premier Biosoft International, Palo Alto, CA) (see Table 1).

**Table 1:** Primers used in this study for RT-qPCR.

GenBank Locus Tag	Gene Name	Primer Sequence (forward and reverse)
	*16S rRNA*	ACCAGAAAGGGACGGCTAAC
		TAGCCTTTTACTCCAGACTTTCCTG
SMU.1004	*gtfB*	AGCAATGCAGCCAATCTACAAAT
		ACGAACTTTGCCGTTATTGTCA
SMU.1568	*malE*	CTATTACCAGCAAGGCAAC
		ACACCAGCATCATTTCCC
SMU.576	*lytT*	TGGCAAGACAAGAGTTAA
		GCTAATATCTTCAGCTTCAA
SMU.1423	*pdhA*	ATGCCAAACTATAAAGATTTAC
		TCTTGGGCTTCAATATCT
SMU.1571	*msmK*	CCTTTATATTGATGATAAACTCA
		CATATTTTCATAAACGCTCAT
SMU.103	*sorA*	ATAACAGGAATGAACTTACC
		ATTTACATACACTAATGATGAAC
SMU.1596	*celD*	GCTGTCATTATTCGCTTT
		CAAATCGAGTAACACCATTA
SMU.1665	*livF*	AAGTCGTTTCTCTTATTGG
		TTAATTTCCCCTGAACTTG
SMU.1489	*lacX*	TCCTGACAAAGAAATGCTGATG
		ACCTGATACTCTGTGCGAATAG
SMU.876	*msmR*	TCACTTAGAAGAGAGCAATAGC
		AACTGCCATACTGCGAATG
SMU.1528c	*atpD*	GGCGACAAGTCTCAAAGAATTG
		AACCATCAGTTGACTCCATAGC
SMU.1955	*groES*	GAAAGAAGAAAAGGAACAAAC
		CACCAACAGCTACTACTT
SMU.1561	*trkB*	CTTATGTGGCTAAGCAATT
		CTCCATGTAAGACCTCAG

Briefly, cDNAs were synthesized using the BioRad iScript cDNA synthesis kit (Bio-Rad Laboratories, Inc., CA). To check for DNA contamination, purified total RNA without reverse transcriptase served as a negative control. The resulting cDNA and negative controls were amplified by a MyiQ qPCR detection system with iQ SYBR Green supermix (Bio-Rad Laboratories, Inc., CA, USA) and specific primers. A standard curve was plotted for each primer set, as described elsewhere [20]. The standard curves were used to transform the quantification cycle (*Cq*) values to the relative number of cDNA molecules. Relative expression was calculated by normalizing each gene of interest to the reference gene 16S rRNA [20].

### Microarray experiments

Whole genomic profiling was conducted using *S. mutans* UA159 microarrays provided by the J. Craig Venter Institute (JCVI). Details about the arrays are available at http://pfgrc.jcvi.org/index.php/microarray/array_description/streptococcus_mutans/version1.html. The biofilms were grown in the presence of 0.5% sucrose, 0.5% sucrose +1% starch, and 1% sucrose, and the RNA was extracted and purified at selected time points (21, 24, 30 and 34 h) [22]. A reference RNA prepared from a single large-scale culture (1 liter) of *S. mutans* UA159 cells that had been grown in BHI broth to an optical density of 0.5 at 600 nm (mid-exponential growth phase) was used in every experiment [24]. The reference RNA was purified as described by Cury and Koo [22], aliquoted, and stored at −80°C. The use of reference RNA as a normalization tool in microarray experiments reduces bias due to dye incorporation, eliminate the need for dye swap, and can be used for multiple comparisons as detailed elsewhere [25]. The experimental and reference RNAs were used to generate cDNA according to the protocol provided by JCVI at http://pfgrc.jcvi.org/index.php/microarray/protocols.html. Purified experimental cDNAs were coupled with indocarbocyanine (Cy3)-dUTP, while reference cDNA was coupled with indodicarbocyanine (Cy5)-dUTP (Amersham Biosciences, Piscataway, NJ). Hybridizations were carried out using the MAUI hybridization system (BioMicro Systems, Salt Lake City, UT). The slides were then washed according to JCVI protocols and scanned using a GenePix scanner (Axon Instruments Inc., Union City, CA) at 532 nm (Cy3 channel) and 635 nm (Cy5 channel).

### Microarray data analysis

After the slides were scanned, single-channel images were loaded into JCVI Spotfinder software (http://www.tm4.org/spotfinder.html) and overlaid. A spot grid was created according to JCVI specifications and then manually adjusted to fit all spots within the grid. The intensity values of each spot were measured and saved into “.mev” files. Data were normalized using LOWESS and standard deviation regularization with default settings, followed by in-slide replicate analysis using the JCVI microarray data analysis software MIDAS (http://www.tm4.org/midas.html). Spots that were flagged as having either low intensity values or low signal saturation were automatically discarded. The statistical analysis was carried out using BRB-ArrayTools (http://linus.nci.nih.gov/BRB-ArrayTools.html) with a cutoff *P* value of 0.001 for class prediction and *P* value of 0.001 and 0.01 for class comparison, both paired. A total of 4 microarray slides pairs were selected by BRB for class comparison analysis. The paired analyzes were: A) 0.5% sucrose +1% starch versus 1% sucrose; B) 0.5% sucrose +1% starch versus 0.5% sucrose; and C) 0.5% sucrose versus 1% sucrose. Thus, 3 sets of data were obtained for each time point.

### Microarray data organization and time course analysis

Due to the complexity of data analyses (sugar concentration and temporal effects), we designed a data mining and organization software named “Microarray Data Visualizer (MDV)” to process the BRB data files. The MDV software was written in Python language, and its GUI part was created with the TKinter GUI toolbox. This tool allows users to: 1) input experimental data to a locally installed MDV; 2) perform a variety of set functions, such as mapping GO numbers, functional class, gene names and pathways for each annotated gene; 3) select groups of genes based on their expression in different conditions; and 4) export results to MS Excel. In addition, all parameters are fully adjustable. The beta release of this tool can be downloaded from the LANL Oralgen site (http://www.oralgen.lanl.gov/). The annotation file of the microarray slides comes with a Unique ID (GenBank Locus Tag number) and description. However, to better understand the biological role of the differentially expressed genes (and correlate with published data), the addition of gene names and the functional class of these genes would be helpful. In this context, the MDV can create additional parameters such as gene name and functional class, and also uses Venn diagram analysis approach to facilitate hypothesis-driven data organization. The use of Venn diagram assisted us in selecting the genes that were detected as differentially expressed for the comparison A (0.5% sucrose +1% starch versus 1% sucrose) and B (0.5% sucrose +1% starch versus 0.5% sucrose), excluding those genes that were detected in the comparison C (0.5% sucrose versus 1% sucrose) (which are not related to starch+sucrose effects). A similar approach was performed for time course analysis to check whether the genes detected in one time point were also detected in other time points evaluated. The files generated by MDV analysis were exported to MS Excel and a cutoff for fold of change of ≥1.8 and ≤0.6 was applied, which correlates well with RT-qPCR validation process based on preliminary experiments.

### Validation of microarray data

Standard RT-qPCR and specific biochemical assays were used to validate the microarray data.

#### Effect of DNAse I on biofilm biomass

*S. mutans* biofilms were formed on sHA as described above. After 30 h of biofilms growth, the biofilms were transferred to UFTYE supplemented (or not) with 50 U/ml DNAse I (TURBO DNase, Ambion, TX). At the end of the experimental period (44 h), biofilms were removed and homogenized by sonication, and aliquots were taken to determine biomass (dry-weight) and microbial counting [26]. The sonication procedures provide homogeneous suspension, which reduces variance in biomass determination, eDNA extraction, and do not lyse the cells as determined experimentally [22].

#### Purification and quantification of extracellular DNA (eDNA)

The extraction, purification and quantification of eDNA were performed according to Rice *et al.* [27]. After 44 h, the biofilms were rinsed in 0.5 M EDTA, and removed into chilled tubes containing 50 mM Tris-HCl/10 mM EDTA/500 mM NaCl, pH 8.0. Biofilms were sonicated [22] and the biofilms suspensions were centrifuged (10 min, 5500 *g*, 4°C). Aliquots of 1 ml of each supernatant was transferred to a tube containing 3 ml of TE buffer (10 mM Tris-HCl/mM EDTA, pH 8.0), and extracted once with an equal volume of phenol/chloroform/isoamyl alcohol (25∶24∶1) and once with chloroform/isoamyl alcohol (24∶1). The aqueous phase of each sample was then mixed with 3 vol of ice-cold 100% (vol/vol) ethanol and 1/10 volume of 3 M Na-acetate (pH 5.2) and stored at -20°C. The next day, the ethanol-precipitated DNA was collected by centrifugation for 20 min at 4°C and 13,000 *g*, washed with ice-cold 70% (vol/vol) ethanol, air-dried, and dissolved in 20 µl of TE buffer. The amount of eDNA was determined spectrophotometrically at 260 nm.

#### Determination of intracellular iodophilic polysaccharides in biofilms

Biofilms were removed after 30 h of growth, and homogenized by sonication as described elsewhere [26]. The homogenized suspension was analyzed for biomass (dry-weight) and intracellular iodophilic (IPS) polysaccharides content. The IPS were extracted with hot 5.3 M KOH (0.8 mg of biofilm dry weight/mL of KOH), and quantified using 0.2% I_2_/2% KI solution and glycogen as standard as described by Koo *et al* [26] and DiPersio *et al*. [28].

### Microarray data accession number

DNA microarray data have been deposited in the NCBI Gene Expression Omnibus (GEO) database (http://www.ncbi.nlm.nih.gov/geo) under GEO Series accession number GSE21831.

### Statistical analyses

An exploratory data analysis of biochemical and RT-qPCR assays was performed to select the statistical test; the assumptions of equality of variances and normal distribution of errors were also checked. The data were then analyzed using ANOVA, and the F-test was used to test for differences among the groups. When significant differences were detected, pairwise comparisons were made between all the groups using Tukey's method to adjust for multiple comparisons. Triplicates from at least three separate experiments were conducted in each of the assays. Statistical software JMP version 3.1 was used to perform the analyses. The level of significance was set at 5%.

## Results

### Selection of experimental groups for microarray experiments

We initially investigated the effects of different concentrations of sucrose alone or in combination with starch on extracellular polysaccharide (EPS) matrix formation and biofilm accumulation on the saliva-coated hydroxyapatite (sHA) surface (Appendix S1). Three experimental groups were selected for *S. mutans*-transcriptome analysis: 0.5% sucrose, 0.5% sucrose+1% starch and 1% sucrose. These groups were selected because 0.5% and 1% sucrose are the minimum and the optimum concentration of the carbohydrate for biofilm development by *S. mutans* using our *in vitro* model. In addition, the combination 0.5% sucrose+1% starch resulted in biofilms with more biomass, higher amounts of insoluble EPS, and increased *gtfB* expression (the gene encoding enzyme for synthesis of water insoluble glucans) than in other biofilms; the presence of starch alone results in negligible biofilm formation.

Concomitantly, the availability of different types and size of sugars (degree of polymerization – DP, and molecular weight - MW) released from starch digestion by the surface-adsorbed salivary α-amylase was also examined overtime (Appendix S1). Starch was progressively digested by the amylase until the enzyme no longer catalyzes the hydrolysis (about 50% of initial starch concentration). The starch hydrolysates released by surface-amylase activity ranged from high (1.45–21.5 kDa with DP varying from 9 to 132.5) to low molecular weight (maltose and maltotriose) products; however, glucose was not detected (Appendix S1).

**Figure 1:**
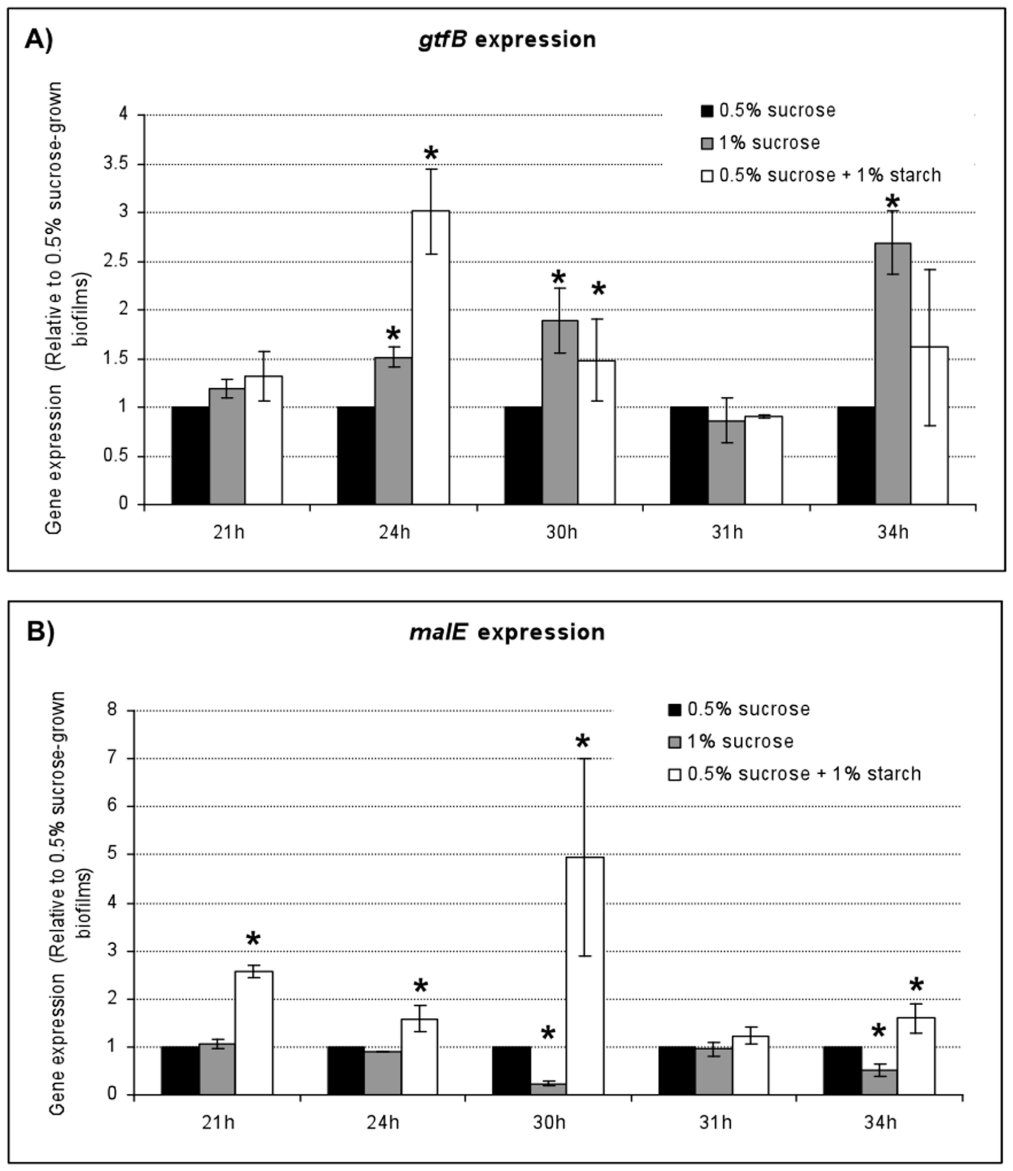
RT-qPCR analysis of *gtfB* (A) and *malE* (B) genes expression. *S. mutans* growing in the presence of 0.5% sucrose, 0.5% sucrose +1% starch, and 1% sucrose at distinct time points of biofilms development process. The mRNA level of *gtfB* and *malE* in each sample was normalized to that of 16S rRNA. These values were then compared to those from 0.5% sucrose-grown biofilms (corresponding to an arbitrary value of 1) to determine the change (*n*-fold) in gene expression. Data are expressed as means ± standard deviations of triplicates from at least three separate experiments. Values marked with an asterisk are significantly different from the value for the 0.5% sucrose-grown biofilms (*P*<0.05, Tukey's test).

Furthermore, we assessed the expression of genes *gtfB* and *malE* (associated with uptake of maltose, which is one of the main starch hydrolysates) by qRT-PCR at distinct stages of biofilm development under the selected carbohydrate sources. These genes were chosen because in our previous study we found that their expression was affected by the combination of sucrose plus starch [16], and are both relevant for biofilm formation (EPS-matrix synthesis) and physiology (intracellular sugar metabolism). A greater differential expression of these genes was observed among the experimental groups at 21, 24, 30 and 34 h of biofilms development (Fig. 1). This output also guided our selection of these 4 time-points for microarray experiments. The pH values of culture medium surrounding the biofilms from the different experimental groups were not significantly different from each other (6.67±0.03 at 21 h, 5.86±0.19 at 24 h, 4.63±0.01 at 30 h, and 4.83±0.03 at 34 h; *P*>0.05).

### Microarray data analysis

#### Data analysis using Microarray Data Visualizer (MDV)

**Figure 2:**
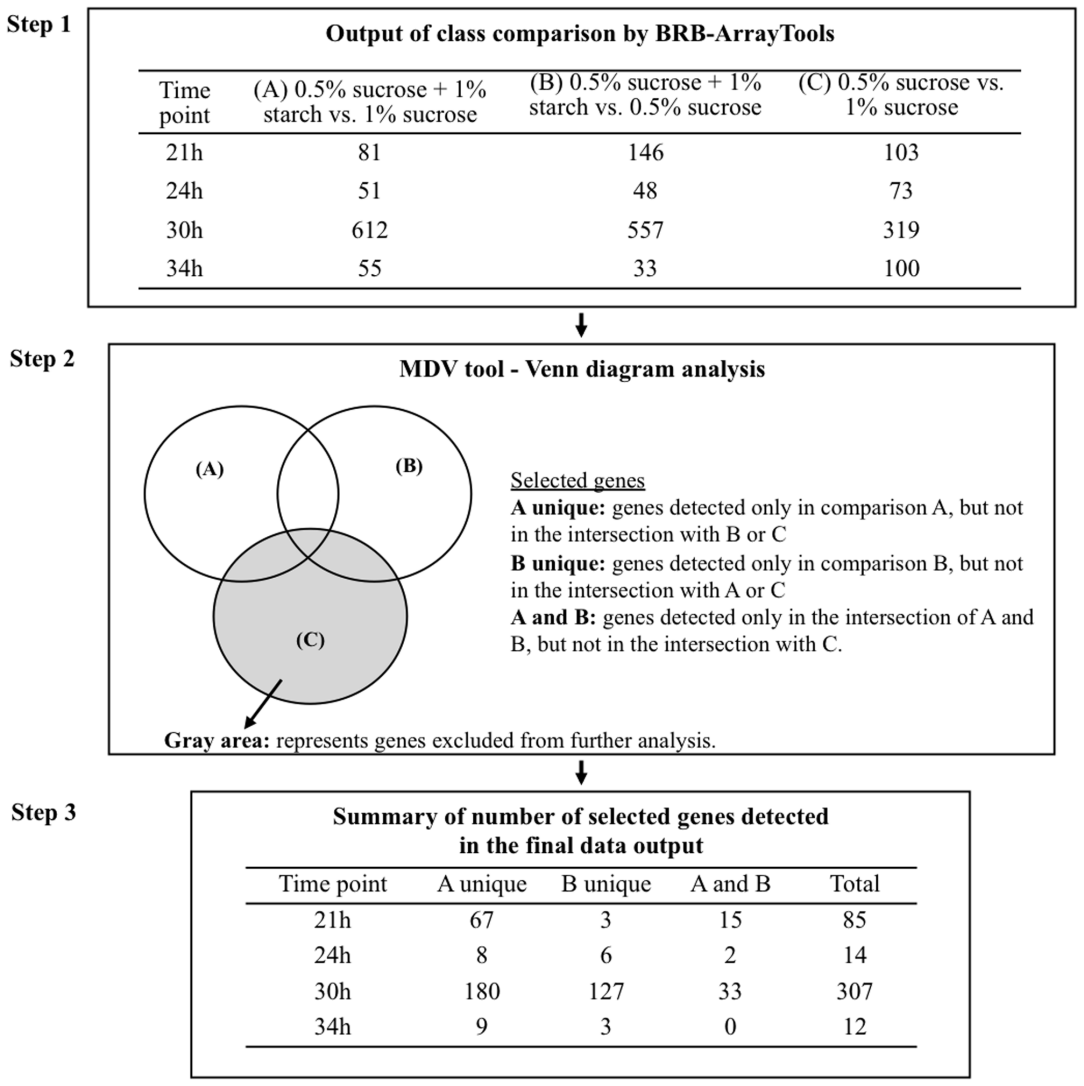
Schematic illustration of the microarray data analysis using BRB-ArrayTools in conjunction with MDV software. Step 1: represent the number of genes detected as differentially expressed in each condition and time point evaluated (see Data S1 ). Step 2: Microarray Data Visualizer (MDV) using the Venn diagram to select genes of interest. Step 3: genes selected according to MDV analysis and cutoff of fold of change in gene expression (fold ≥1.8 and ≤0.6; see Data S2).

The following comparisons (for each time point) were examined: A) 0.5% sucrose +1% starch vs. 1% sucrose, B) 0.5% sucrose +1% starch vs. 0.5% sucrose, and C) 0.5% sucrose vs. 1% sucrose; this strategy allowed us to distinguish the effects caused by sucrose+starch from those associated with distinct concentrations of sucrose. The microarray data from the multiple comparisons generated a large data output using standard procedures with BRB-ArrayTools (Data S1 – original array data; Data S2 – quality scores of the arrays). To streamline our transcriptome analysis, we developed a new data mining and organization software, Microarray Data Visualizer (MDV). The data processing and analysis were done in three steps as shown in Fig. 2.

**Figure 3:**
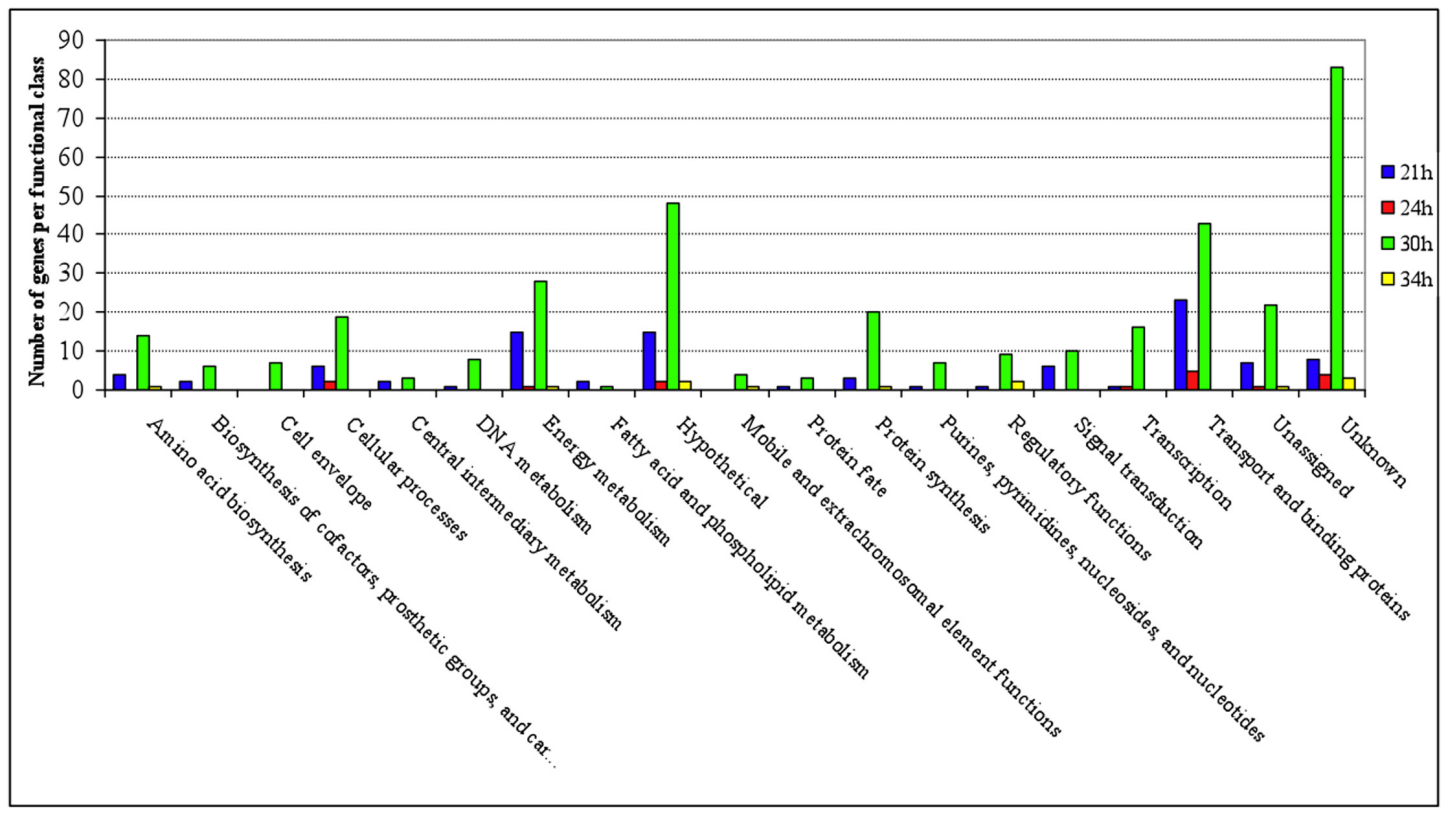
Number of *S. mutans* genes differentially expressed in suc+st-biofilms (vs. suc-biofilms) at various time-points organized by functional class. Suc+st-biofilms were formed in 0.5% sucrose+1% starch; suc-biofilms were formed in 1% sucrose. Gene annotations are based on information provided by the Los Alamos National Laboratory (www.oralgen.lanl.gov) or by published literature available at the same Website.

The raw data analyzed by BRB-ArrayTools (Step 1, Fig. 2) was further processed by MDV using the Venn diagram (Step 2, Fig. 2) to select and filter the genes of interest according to our working hypothesis: that sucrose and starch combination triggers specific transcriptional response associated with enhanced virulence (cariogenicity) of *S. mutans* in biofilms. In our study, the groups of genes related to our hypothesis are those differentially expressed in comparison A (0.5% sucrose +1% starch vs. 1% sucrose) and B (0.5% sucrose +1% starch vs. 0.5% sucrose), but not the genes in comparison C (0.5% sucrose vs. 1% sucrose) (Step 2, Fig. 2). Moreover, this analysis also excluded genes that were detected simultaneously in the 3 comparisons (A and B and C), because these are not uniquely related to the influence of sucrose and starch in combination. All the genes excluded from further analysis are represented in gray in Fig. 2 (Step 2). Then, the data was collected and organized for further examination (Step 3, Fig. 2 and Data S3 – post-MDV analysis). MDV greatly facilitated the mining of the large and complex data sets from our microarray experiments, which reduced the total number of genes to be analyzed and at the same time filtered-out the genes not directly related to starch+sucrose effects. Another important feature of this software is the automated organization of the selected genes according to NCBI gene names and functional classes, which facilitates the visualization and organization of the microarray data (Fig. 3; Data S3). The MDV software could be particularly helpful to other biofilm-related fields using whole-genome profiling for comparison of multiple experimental conditions, such as comparative-transcriptome of distinct strains or in response to therapeutic agents.

**Figure 4:**
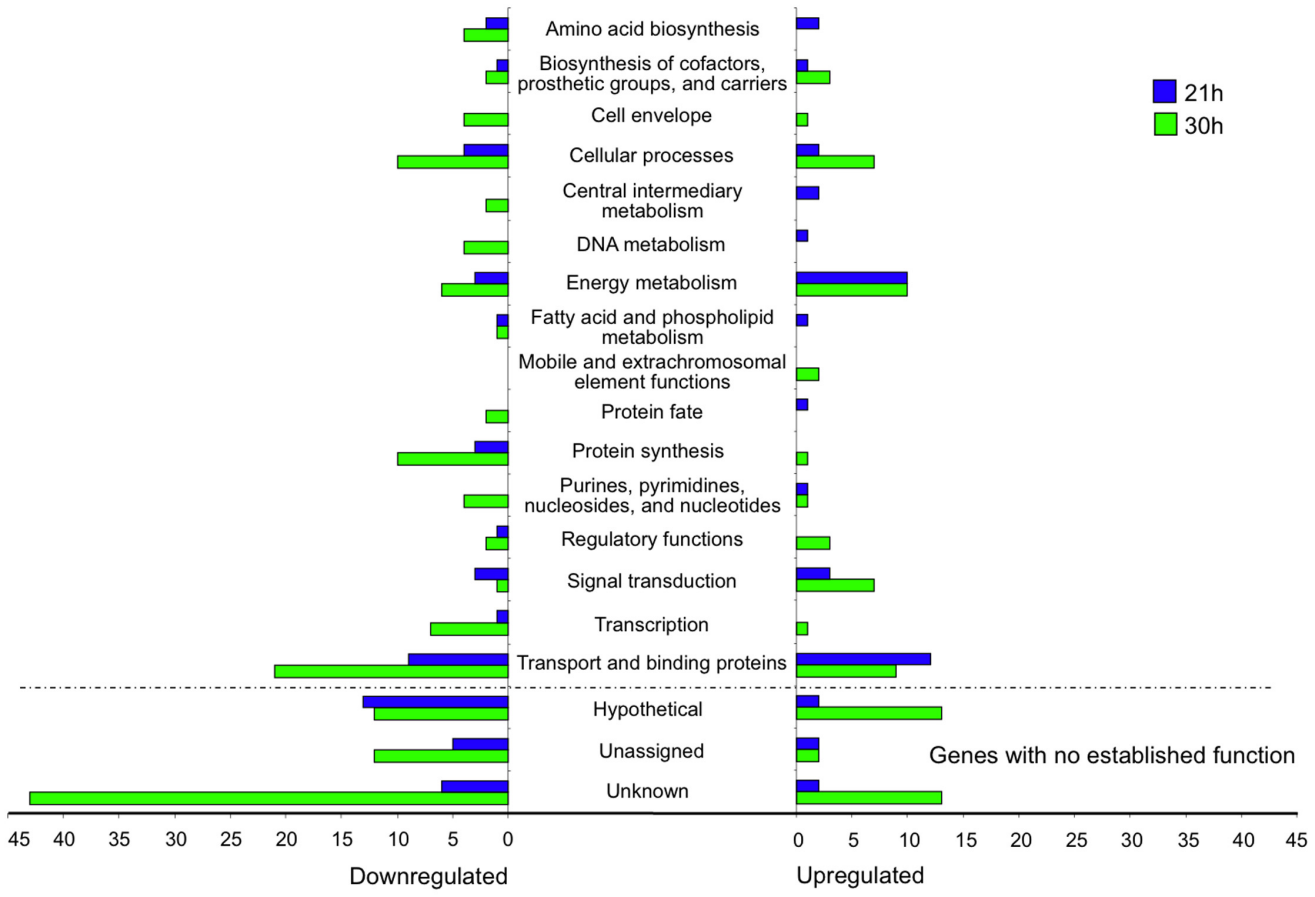
Differential regulation of *S. mutans* gene expression in suc+st-biofilms at 21 and 30 h. Genes were classified into 19 main functional classes (some genes have more than one functional class). Bars indicate the numbers of genes whose expression was modified at 21 h (blue bars) and 30 h (green bars). On the left, genes that were downregulated in suc+st-biofilms (vs. suc-biofilms): 52 and 147 genes for 21 h and 30 h, respectively. On the right, genes that were upregulated in suc+st-biofilms (vs. suc-biofilms): 42 and 73 genes for 21 h and 30 h, respectively. Suc+st-biofilms were formed in 0.5% sucrose +1% starch; suc-biofilms were formed in 1% sucrose. Gene annotations are based on information provided by the Los Alamos National Laboratory (www.oralgen.lanl.gov) or by published literature available at the same Website.

MDV analysis revealed that most of the genes differentially expressed were detected at 21 h and 30 h of biofilm development process, and in the comparison A (0.5% sucrose +1% starch vs. 1% sucrose) (Fig. 2, Step 3). Considering that about 50% of starch is digested by surface-adsorbed amylase present in sHA, the data from comparison A may be biologically more relevant because the total amount of sugar available for bacterial metabolism would be similar. Therefore, we focused on the comparison 0.5% sucrose +1% starch (suc+st) vs. 1% sucrose (suc) biofilms at 21 and 30 h for a more detailed and comprehensive analyses (See tables 2, 3 and Fig. 4). The microarray data was validated by RT-qPCR, and all genes selected displayed the same trends observed in the microarrays (Tables 2 and 3).

**Table 2:** Selected genes with known function detected up- and downregulated in 21 h-old biofilms (suc+st vs. suc) after MDV analysis*.

Gene ID**	Gene Name	Fold change		Functional class annotation
		Microarray	qPCR (Avg ± SD)	
SMU.99	*fbaA sorG*	0.6		Energy metabolism; Glycolysis/gluconeogenesis
SMU.104	*yicI*	0.5		Signal transduction; PTS
SMU.114	*-*	1.9		Signal transduction; PTS
SMU.115	*-*	2.3		Signal transduction; PTS
SMU.128	*adhB acoB*	2.9		Energy metabolism; Fermentation
SMU.129	*adhC yugF acoC*	2.8		Energy metabolism; Glycolysis/gluconeogenesis
SMU.183	*sloB*	0.4		Transport and binding proteins; ABC Superfamily: membrane spanning permease
SMU.184	*sloC*	0.3		Transport and binding proteins; ABC Superfamily: substrate-binding protein; Cations and iron carrying compounds
SMU.561	*mutT*	2.0		DNA metabolism; DNA replic., recomb., and repair
SMU.877	*aga*	1.8		Energy metabolism; Sugars - melibiase
SMU.881	*gtfA*	2.1		Energy metabolism; Sugars
				Transport and binding proteins; Carbohydrates, organic alcohols, and acids
SMU.882	*msmK*	3.1		Energy metabolism; Sugars
				Transport and binding proteins; ABC Superfamily: ATP-binding protein
SMU.886	*galK*	1.9		Energy metabolism; Sugars
SMU.887	*galT*	1.8		Energy metabolism; Sugars
SMU.1561	*trkB*	2.2	2.0±0.3	Transport and binding proteins; Cations and iron carrying compounds
SMU.1563	*pacL*	2.0		Transport and binding proteins; Cations and iron carrying compounds
SMU.1564	*glg*	7.4		Energy metabolism; Sugars
SMU.1565	*malM*	4.9		Energy metabolism; Sugars
SMU.1569	*malF malC*	3.1		Transport and binding proteins; ABC Superfamily: membrane spanning permease
SMU.1570	*malG malX*	2.7		Transport and binding proteins; ABC Superfamily: membrane spanning permease
SMU.1571	*msmK*	2.9	1.8±0.2	Transport and binding proteins; ABC Superfamily: ATP-binding protein
SMU.1841	*scrA*	0.6		Signal transduction; PTS
SMU.1879	*manN ptnD*	0.6		Signal transduction; PTS
				Transport and binding proteins; Carbohydrates, organic alcohols, and acids
SMU.1954	*groEL*	2.0		Cellular processes; Chaperones
SMU.1955	*groES*	2.5	4.6±0.7	Cellular processes; Chaperones
SMU.2047	*ptsG ptaA*	2.0		Energy metabolism; Sugars

*suc+st −0.5% sucrose +1% starch; suc −1% sucrose;

**Organized by numerical order.

**Table 3:** Selected genes with known function detected up- and downregulated in 30 h-old biofilms (suc+st vs. suc) after MDV analysis*.

Gene ID**	Gene Name	Fold change		Functional class annotation
		Microarray	qPCR (Avg ± SD)	
SMU.101	*sorC*	16.8		Signal transduction; PTS
SMU.103	*sorA*	15.8	31.4±16.1	Signal transduction; PTS
SMU.104	*yicI*	17.7		Signal transduction; PTS
SMU.183	*sloB*	0.6		Transport and binding proteins; ABC Superfamily: membrane spanning permease
SMU.186	*sloR*	0.6		Cellular processes; Pathogenesis
SMU.480	*priA*	0.6		DNA metabolism; DNA replic., recomb., and repair
SMU.576	*lytT*	8.7	9.6±5.1	Signal transduction; Two-component systems
SMU.644	*coiA*	0.6		Cellular processes; Competence
SMU.672	*citC icd*	0.4		Energy metabolism; TCA cycle
SMU.675	*ptsI*	1.8		Signal transduction; PTS
SMU.876	*msmR*	2.6	6.2±2.8	Regulatory functions; Two component system; Transport and binding proteins; Carbohydrates, organic alcohols, and acids
SMU.928	*kinF*	0.6		Signal transduction; Two-component systems
SMU.980	*bglP ptbA*	3.6		Transport and binding proteins; Carbohydrates, organic alcohols, and acids
SMU.995	*yclN*	0.6		Transport and binding proteins; ABC Superfamily: membrane spanning permease
SMU.1043	*pta*	3.3		Energy metabolism; Fermentation
SMU.1075	*dfp dpfA*	1.9		Biosynthesis of cofactors, prosthetic groups, and carriers; Pantothenate and coenzyme A
SMU.1117	*nox-2 naoX*	2.2		Energy metabolism; Glycolysis/gluconeogenesis
SMU.1126	*coaA*	2.0		Biosynthesis of cofactors, prosthetic groups, and carriers; Pantothenate and coenzyme A
SMU.1191	*pfk pfkA*	0.6		Energy metabolism; Glycolysis/gluconeogenesis
SMU.1247	*eno enoA*	0.6		Energy metabolism; Glycolysis/gluconeogenesis
SMU.1423	*pdhA acoA*	29.2	56.0±3.2	Energy metabolism; Fermentation
SMU.1489	*lacX galM*	2.9	3.4±1.1	Energy metabolism; Glycolysis/gluconeogenesis
SMU.1491	*lacE*	3.9		Signal transduction; PTS
SMU.1493	*lacD*	3.4		Energy metabolism; Sugars
SMU.1561	*trkB*	1.9	1.8±0.3	Transport and binding proteins; Cations and iron carrying compounds
SMU.1563	*pacL*	2.1		Transport and binding proteins; Cations and iron carrying compounds
SMU.1566	*malR rliA*	2.1		Regulatory functions; DNA interactions
SMU.1571	*msmK*	26.1	48.6±15.3	Transport and binding proteins; ABC Superfamily: ATP-binding protein
SMU.1591	*ccpA regM*	2.0		Regulatory functions; DNA interactions
SMU.1596	*celB lacE*	13.4	6.5±2.5	Signal transduction; PTS
SMU.1665	*livF*	0.6	0.5±0.2	Transport and binding proteins; ABC Superfamily: ATP-binding protein
SMU.1843	*scrB*	1.8		Energy metabolism; Sugars
SMU.1983	*comYD cglD*	0.5		Cellular processes; DNA transformation

*suc+st −0.5% sucrose +1% starch; suc −1% sucrose;

**Organized by numerical order.

#### Transcriptome response of biofilms at 21 h

At this stage of biofilm formation, 82 genes were detected as differentially expressed in suc+st-biofilm (vs. suc-biofilm). Among them 52 genes had assigned function whereas 30 had no established function (grouped as unknown, unassigned or hypothetical) (Fig. 4). Several known genes differentially expressed in suc+st-biofilm were related to sugar metabolism, such as upregulation of maltose/maltotriose uptake genes and downregulation of mannose, sorbose and sucrose transport systems. Those genes represent 26.9% of the genes with assigned function and were distributed into 3 functional classes: energy metabolism, transport and binding proteins, and signal transduction – PTS (Fig. 4, Table 2 and Data S3); most of the genes related to energy metabolism were upregulated (Fig. 4, Table 2).

Other genes induced at this time point include those involved in DNA repair (*mutT*), in osmotic stress response (*pacL* and *trkB*), and the chaperones *groEL* and *groES*. The upregulation of *pacL*, *trkB*, *groEL* and *groES* indicate that the presence of starch and starch hydrolysates may cause environmental stress to *S. mutans* biofilms-cells.

#### Transcriptome response of biofilms at 30 h

A total of 213 genes were detected as differentially expressed in suc+st-biofilm (vs. suc-biofilm) at 30-h of development; among them 95 genes had no established functions (Fig. 4). In contrast to 21 h biofilms, only 11.9% of the genes with known function (detected in suc+st-biofilm) were related to sugar uptake and transport, including upregulation of genes for sucrose, lactose, cellobiose, multisugar transport system (*msm* operon) and maltose/maltotriose uptake, and downregulation of genes from fructose and trehalose transport systems. The other genes (88.1%) were distributed into 15 functional classes (Fig. 4). For example, there was upregulation of biosynthesis of cofactors for coenzyme A, fermentation (*pdhA*), glycolysis (*naoX*), potassium uptake (*pacL*, *trkB*) and two component system (TCS) - *lytT*; whereas DNA repair (*priA*, *ogt*, *dnaC*), DNA transformation (*comYD*, *comX1*, *mecA*, *coiA*), TCS (*kinF*), cell pathogenesis (*sloBC*), TCA cycle (*citBZC*) and iron transport *(yclNPQ* operon) genes were downregulated (Data S3; selected genes are shown in Table 3).

Furthermore, the data outcome also showed that 23 genes were detected in more than one time point of biofilm development (see details on time course analysis section of Data S3). No genes were differentially expressed across the 4 time points evaluated. Only two genes, SMU.1067 (a putative ABC transporter) and *msmK* (SMU.1571; maltose uptake), were detected in biofilms at 21, 24 and 30 h. From those 23 genes, 12 were detected in both 21 and 30 h, and 9 of them had a similar trend of fold of gene expression. For example, the genes related to osmotic stress response *trkB* and *pacL* were upregulated at 21 and 30 h.

It is noteworthy that the number of genes detected as differentially expressed in suc+st-biofilms that had no established function increased as the biofilms matured. At 21 h, 36.6% of the genes detected had no established function, and at 30 h, the number of genes with uncharacterized function increased to 44.6%. Among those genes with unknown function, the gene SMU.2026 was upregulated 18-fold at 30 h in suc+st- vs. suc-biofilm (Data S3), which may be related to metabolism of maltose transported into the cell by MalT (PtsG) as recently suggested [12]. The genes *malE* and *gtfB* were not detected as differentially expressed in the microarrays after analysis with MDV. These apparent discrepant results are likely due to differences in sensitivity between microarray and qRT-PCR technologies. qRT-PCR that uses gene-specific primers for amplification is an extremely sensitive assay for detecting changes in gene expression, whereas microarrays are comparatively less sensitive (albeit more comprehensive). Therefore, the qRT-PCR results in fact complement our microarray analysis. Moreover, while *malE* was not detected in the microarrays, other genes in the same operon were detected (genes *malF*, *malG* and *msmK*).

Considering the data from our previous studies [15], [16] and the current transcriptomic analysis, we conducted further biochemical assays to explore the upregulation of two specific genes that may be associated with the establishment of extracellular matrix and acid production by *S. mutans* within biofilms: *lytT* (SMU.576) and *glg* (SMU.1564).

### Biochemical assays

#### *lytT* gene up-regulation and influence of extracellular DNA (eDNA) on biofilm biomass

**Figure 5:**
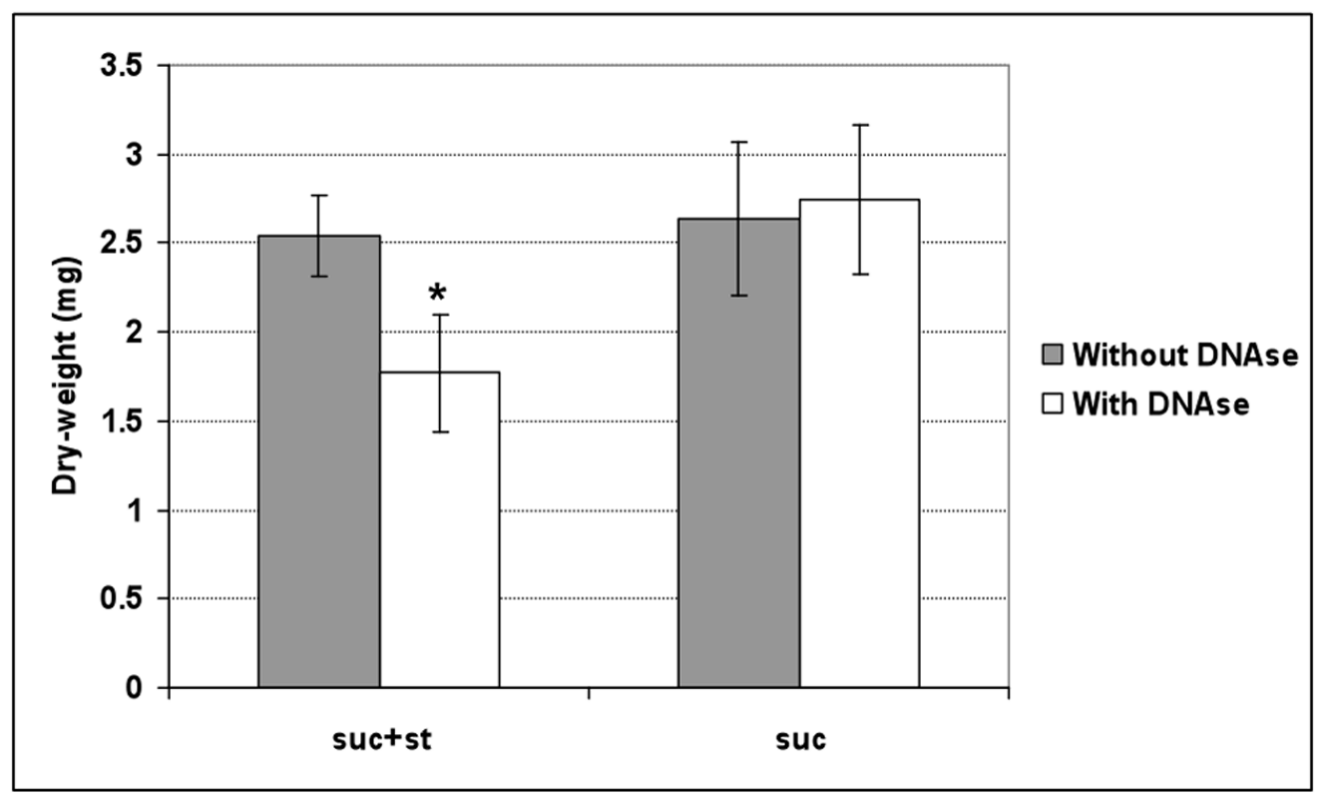
Influence of DNase I treatment on the biomass (dry-weight) of the biofilms. Amount of dry-weight of biofilms (at 44 h) treated (With DNAse I) or not with DNase I (Without DNAse). The data shown are mean values (±SD; n = 9) from three independent experiments. Values with an asterisk (*) are significantly different from other groups (*P*<0.05, ANOVA comparison for all pairs using Tukey test); suc+st −0.5% sucrose +1% starch, and suc −1% sucrose. The numbers of CFU recovered from biofilms treated (or not) with DNAse I were: 5.3±1.6×10^8^ (4.9±1.3×10^8^) for suc+st-biofilms, and 1.9 ±0.4×10^8^ (2.9±1.3×10^8^) for suc-biofilms. The biomass (dry-weight) values are lower from those presented in the Appendix S1 because of the differences of the biofilms age (44 h vs. 120 h).

The *lytT* gene is associated with bacteria autolysis [29], [30] and was upregulated in suc+st-biofilms at 30 h; the lyses process release DNA to the extracellular environment. Considering that eDNA is implicated in the formation and stability of the extracellular matrix [27], [31], [32], we investigated whether treatments with DNAse I at this time-point would affect further formation and accumulation of biofilms in the presence of starch and sucrose. The addition of 50 U of DNAse I in the culture media resulted in 30.2% decrease of biomass of suc+st-biofilms (vs. non-treated biofilms; *P*<0.05) whereas the presence of DNAse I had no significant effect on biomass of biofilms grown in sucrose alone at 44 h of development (*P*>0.05; Fig. 5). The addition of DNAse I did not affect the number of viable cells in the biofilms as determined by counting the number of colony forming unit per biofilm. Finally, the amount of eDNA recovered from suc+st-biofilms was significantly higher than the amount recovered from suc-biofilms (9.5±2.7 µg/mg dry- weight and 6.9±2.9 µg/mg, respectively; *P*<0.05).

#### *glg* gene upregulation and effects on intracellular polysaccharide storage (IPS)

**Figure 6:**
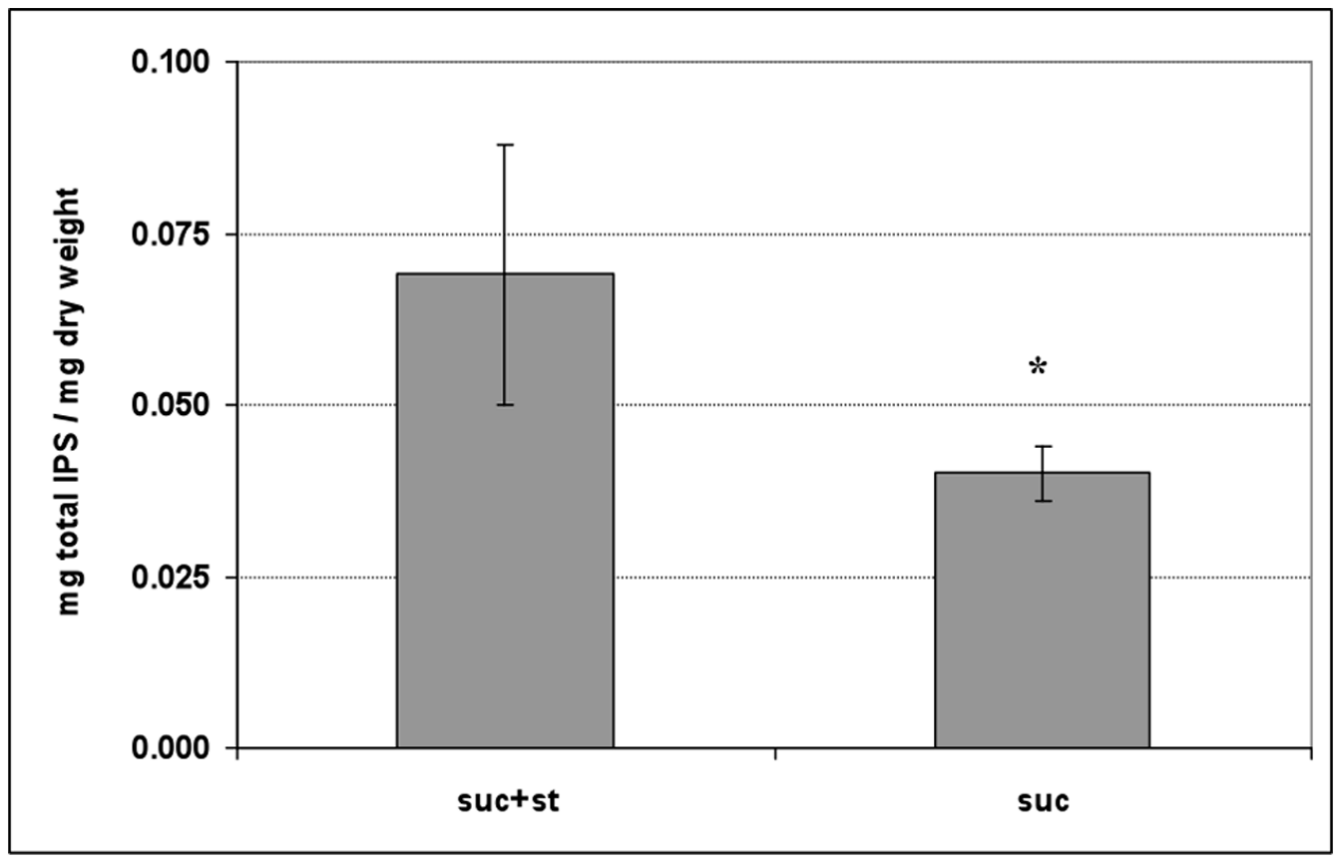
Amounts of intracellular polysaccharides (IPS) in the biofilms. Total amounts of IPS in mg/mg of biofilm dry-weight. The data sh own are mean values (±SD; n = 9) from three independent experiments. Values with an asterisk (*) are significantly different from other group (*P*<0.05, ANOVA comparison for all pairs using Tukey test); suc+st −0.5% sucrose +1% starch, and suc−1% sucrose.

The expression of gene *glg* (which encodes a glycogen phosphorylase) was enhanced in biofilms formed in the presence of sucrose plus starch at 21 h and 24 h (vs. suc-biofilms; see Data S3) which could reflect in changes on IPS metabolism during the biofilms development process. Indeed, the determination of the IPS content revealed that suc+st-biofilms at 30 h of biofilm growth have significantly higher amounts of IPS compared to biofilms formed in sucrose alone (*P*<0.05) (Fig.6).

## Discussion

The appearance of biofilms on the tooth surface is associated with complex host-bacterial interactions with dietary constituents found in the oral cavity, which may also modulate the development of pathogenic biofilms related to the disease of dental caries. The combination of sucrose and starch is highly cariogenic *in vivo* [6]–[8], and their simultaneous consumption may be linked with caries activity in adolescent population [33]. The interactions of starch and sucrose with salivary α-amylase and streptococcal Gtfs could enhance the formation and virulence of biofilms by modulating exopolysaccharides synthesis, sugar metabolism and acidogenicity of *S. mutans* [10], [14], [15], [16]. To further advance our previous findings, we conducted a detailed and comprehensive global transcript analysis in biofilms at distinct developmental stages using cDNA microarray in conjunction with a computational tool (MDV) for data mining.

The results indicate that the presence of starch and sucrose resulted in dynamic remodeling of the transcriptome of *S. mutans* within biofilms, which may be related to changing environmental conditions associated with gradual digestion of starch by amylase in the salivary pellicle (as would occur *in vivo*). Initially, the hydrolysates are mostly large oligosaccharides with average DP and MW of 132.5 and 21.5 kDa (after 1 h digestion) that are further digested to 14.4 kDa (between 1–2 h) and then to smaller 1.45 kDa oligomers. Concomitantly, the proportions of maltose and maltotriose rapidly increase overtime; sucrose, of course is readily available for bacterial metabolism in the culture medium. The 10 to 20 kDa oligosaccharides present at the early stages of starch digestion can be incorporated during glucan synthesis by GtfB in the presence of sucrose through acceptor-reactions [9], [10], [34]. The presence of additional acceptors could be sensed by *S. mutans* resulting in upregulation of *gtfB* in suc+st-biofilms since the starch oligomers act as primers for glucan synthesis by GtfB but not for other Gtf enzymes [10], which corroborates with lack of effect on *gtfC* and *gtfD* expression [16]. The increased synthesis of GtfB-type insoluble glucans triggers the formation of microcolonies by *S. mutans* [21], which increase the coherence and thickness of the biofilms, and thereby influencing their architecture, diffusion properties and pathogenicity [16], [21], [23], [35].

Conversely, the increasing availability of maltose and maltotriose released from continuous starch hydrolysis could explain the elevated levels of expression of genes related to maltose uptake (e.g. *malE*, *msmK*) as the biofilms transit from 21 to 30 h of development. The increased availability of metabolizable carbohydrates contributes with the acidification of the suc+st-biofilms overtime which may also influence the expression of *gtfB*. Furthermore, as the environmental pH falls due to acid production by *S. mutans* and glucans are synthesized, the pathways required for optimal metabolism and survival of *S. mutans* in biofilms may be switched leading to extensive changes of the gene expression profile at 30 h of biofilms formation when compared to other time-points.

The microarray analysis identified four major themes by which *S. mutans* in biofilm-mode responds to changing environmental conditions *in situ* as a result of the interplay of host-diet-bacterial factors: (1) modulation of genes associated with extracellular matrix assembly-development, (2) sugar uptake and glycogen metabolism, (3) stress responses and (4) regulation of a large number of uncharacterized genes. Here, we focused our data analysis on sucrose and starch influences on extracellular matrix development and sugar metabolism in order to connect with our previous findings [15], [16], and because these processes are critical for *S. mutans* virulence.

### *lytT* and extracellular matrix formation in the presence of sucrose and starch

Our data indicate that upregulation of *lytT* may be associated with release of eDNA and its potential role on the development of extracellular matrix in suc+st-biofilms. The *lytT* gene encodes for the response regulator of the LytST TCS and has been implicated in bacterial autolysis [29], [30] and subsequent release of DNA to the extracellular environment. The TCS LytST (encoded by genes SMU.576 and SMU.577) is required for the activation of expression of *lrgAB* genes, which are part of *S. mutans* arsenal to control autolysis and biofilm formation, and may be regulated according to availability of different carbohydrates sources via CcpA (carbon catabolite protein A) [30]. The *ccpA* gene was also upregulated in suc+st-biofilm at 30 h indicating that the presence of undigested starch, starch hydrolysates and sucrose may be modulating autolysis (and DNA release), in part through simultaneous expression of *lytT* and *ccpA* genes.

Notably, eDNA was established as a critical structural component of biofilm matrix for several bacterial pathogens, including *Pseudomonas aeruginosa* [27], [31], [32]. We found that the presence of DNAse I significantly disrupted the biomass and further accumulation of the biofilm in the presence of sucrose and starch (but not in sucrose grown-biofilm). The data agree well with previous findings showing that eDNA enhances *S. mutans* adhesion, surface aggregation and strengthens the matrix [36], [37]. Clearly, eDNA from *S. mutans* may play an important role in the establishment and integrity of extracellular matrix of suc+st-biofilms.

The combined effects of starch and sucrose on DNA release and enhanced synthesis of GtfB-insoluble glucans could explain the formation of a distinctive extracellular matrix in suc+st-biofilms [21], which display more cross-linked (web-like) exopolysaccharides tightly attached to bacterial cells (vs. suc-biofilms). The eDNA could be incorporated during extracellular matrix development by binding to bacterial cell and to exopolymers [36], [37], possibly bridging them and allowing further glucan deposition. The interplay between *lytT* and *gtfB* may explain the formation of a thicker and highly cohesive biofilms containing large microcolonies enmeshed in EPS-rich matrix in the presence of suc+st (vs. sucrose grown-biofilms) at later stages of development [21]. Such structural organization could create chemical gradients because of the differential diffusion of nutrients, metabolic products and oxygen, affecting the microenvironmental conditions in the biofilms [38], [39].

Our data show that suc+st-biofilm at 30 h of development may be under decreased O_2_ availability for *S. mutans* cells as indicated by downregulation of TCA cycle genes (*citBZC*), which are transcriptionally repressed under anaerobic conditions [40]. In addition, the downregulation of these genes favors the fermentation process, as shown by the upregulation of *pdhA*. In *S. mutans*, *pdhA* expression responds to conditions favorable to heterofermentation, and may be associated with aciduricity of this bacterium [40]. Moreover, it has been established that high insoluble glucan content in the EPS matrix acts as a diffusion barrier, trapping acid near the tooth surface and thereby increasing the extent of the acidification period [41], [42] and the cariogenicity of human plaque [43]. Consequently, *S. mutans* growing in the presence of sucrose and gradual availability of starch hydrolysates may display enhanced aciduricity/growth efficiency as the redox potential and oxygen levels fall, and low pH microenvironments are created over the course of biofilm maturation [44]. We are currently examining the exact location of eDNA in the matrix, and how the interplay between *gtfB* and *lytT* affect the development and diffusion properties of the extracellular matrix in suc+st-biofilms.

### *glg* may be linked with enhanced intracellular polysaccharide storage in suc+st-biofilms

We observed an association between sugar uptake and intracellular polysaccharide (IPS) storage, possibly connecting the upregulation of gene *glg* with increased amount of IPS in biofilms formed in the presence of starch and sucrose. Although the exact role of *glg* in *S. mutans* physiology is unknown, this gene encodes a glycogen phosphorylase, an enzyme that has been implicated in IPS formation [45], [46]. This finding is clinically relevant because this glycogen-like storage polymer is important for *S. mutans* virulence and is associated with the pathogenesis of dental caries [45]–[47]. The IPS provide *S. mutans* with endogenous source of carbohydrates which can be metabolized when exogenous fermentable substrate have been depleted in the oral cavity; as a result, IPS can promote the formation of dental caries in animals and humans by prolonging the exposure of tooth surfaces to organic acids and a concomitant lower fasting pH in the matrix of the plaque-biofilm [45]–[47]. Also, the reduction of IPS by therapeutic agents effectively reduced cariogenicity of *S. mutans in vivo* [19], [48]. Thus, the increased accumulation of IPS in addition to changes of the extracellular matrix composition-structure would contribute to the overall acidification at the biofilm-tooth enamel interface. Further studies shall elucidate the precise mechanisms by which starch and sucrose modulate IPS accumulation considering that genes of *glgPADCB* operon (also involved with IPS metabolism) were not detected as differentially expressed in suc+st-biofilms suggesting a different pathway.

In addition to modulating these critical processes, the simultaneous presence of sucrose, undigested starch and varying types/amounts of starch hydrolysates may also act as environmental stressors for *S. mutans* (osmotic stress), as shown by upregulation of *trkB* (potassium uptake protein B) and *pacL* (cation-transporting P-ATPase) genes [49], [50]; the activation of potassium uptake, for example, is related to an increase in medium osmolarity [50]. By triggering these responses, *S. mutans* can efficiently cope and rapidly adapt to changing conditions during the biofilm development observed in our model. This trait is highly relevant in the oral cavity, a dynamic habitat where the bacteria must deal with constant variations in the local environment conditions dictated by extrinsic and intrinsic factors (e.g. exposure of nutrients from host diet) [2], [51].

Clearly, the unique interaction of host- and bacterial-derived enzymes with dietary carbohydrates (sucrose and starch) resulted in extensive remodeling of *S. mutans*-transcriptome over the course of biofilm formation. These complex changes may induce the development of pathogenic biofilms by at least four interconnected ways: 1) increasing production of insoluble glucans and release of DNA which may be acting in concert for the development of a structurally cohesive extracellular matrix; 2) inducing the accumulation of IPS; 3) increasing activation of sugar uptake transport systems (e.g. maltose and maltotriose) which can be further metabolized into acids; and 4) modulating the expression of genes associated with osmotic stress, TCA cycle and fermentation. The combined effects would result in biofilms with increased biomass with low oxygen and highly acidic environment that are cohesive and tightly adherent to the surface, thereby enhancing *S. mutans* survival/persistence and cariogenicity.

Overall, our data provided new information about the remarkable plasticity of the transcriptome of *S. mutans* and its adaptive response to changing environmental conditions within biofilms, which is the mode of growth associated with virulence of this bacterium in the oral cavity. Our comprehensive analysis may provide new leads for molecular pathogenesis research with *S. mutans*, especially in the light of the large number of genes with unidentified function that may reveal new metabolic pathways and/or virulence factors associated with cariogenic biofilm formation. Further studies using both parental and/or mutant strains of *S. mutans* in the presence of α-amylase binding organisms (e.g. *S. gordonii*) should elucidate the exact role of the uncharacterized genes on their ability to survive and compete in a multispecies system.

## Supporting Information

Appendix S1 Appendix S1 presents (i) the experimental process for the selection of experimental groups for the transcriptomic analysis and (ii) determination of starch hydrolysates produced by surface-adsorbed amylase activity.
(0.20 MB DOC)

Data S1 The file shows the microarray data output after using BRB-Array-Tools (pre-MDV analysis).
(0.33 MB XLS)

Data S2 The file shows quality scores of the arrays.
(0.04 MB XLS)

Data S3 The file shows the microarray data output after using BRB-Array-Tools, and then processed with MDV software (post-MDV analysis).
(0.10 MB XLS)
