# Supplementary material for: Dynamics of Streptococcus mutans Transcriptome in Response to Starch and Sucrose during Biofilm Development
Source: PLoS One. 2010 Oct 19;5(10):e13478. doi: 10.1371/journal.pone.0013478 (PMC2957427; doi:10.1371/journal.pone.0013478)
Supplement: Appendix S1 — presents (i) the experimental process for the selection of experimental groups for the transcriptomic analysis and (ii) determination of starch hydrolysates produced by surface-adsorbed amylase activity. (0.20 MB DOC) [file pone.0013478.s001.doc]

**APPENDIX 1**

In this preliminary study, we examined the influence of different concentrations of sucrose, and the effects of addition of different amounts of starch in a given concentration of sucrose on formation of exopolysaccharides (EPS) matrix and biofilms accumulation by *S. mutans* UA159. Furthermore, we also determined the type and size of the sugars released from starch digestion by surface-adsorbed salivary amylase over-time. Biofilms of *S. mutans* were formed on amylase-active saliva coated hydroxyapatite (sHA) discs in batch culture as described in Materials and Methods. First, the biofilms were grown in the presence of several concentrations of sucrose as follows: 0.25, 0.5, 0.75, 1.0, 2.0 and 5% (w/v) sucrose. The biofilms were analyzed for biomass (dry-weight), total amounts of protein, extracellular insoluble and soluble glucans as described elsewhere [15, 20]. Furthermore, the *gtfB* expression in the biofilms was determined using RT-qPCR [18]; *gtfB* is a critical gene for sucrose-dependent biofilms formation and proven virulence factor associated with the pathogenesis of dental caries [17, 18].

**Distinct sucrose concentrations.** The biomass and biochemical composition of the sucrose-grown biofilms are show in the Table A1. The dry-weight, protein and polysaccharides amounts increased as the concentration of sucrose augmented from 0.25% to 1%, showing a positive correlation (R^2^ > 0.8). However, further increase in sucrose concentration did not increase, and actually diminished the biomass and the amounts of protein and insoluble polysaccharides (INS). Interestingly, the amounts of water soluble polysaccharides were enhanced, indicating that concentrations of sucrose higher than 1% induce synthesis of soluble glucans by *S. mutans* rather than insoluble type. The INS (comprised mostly of insoluble glucans) are the main component of the EPS matrix, and are primarily synthesized by *gtfB*. The INS data correlate well with the pattern of *gtfB* expression (Fig. A1), and with the biomass of the biofilms. Sucrose concentration at 0.5% appears to be the turning point for *gtfB* expression in *S. mutans* biofilms, reaching its peak at 1%. Therefore, 1% sucrose was the concentration for maximum INS formation and biofilm accumulation on the sHA surface whereas 0.5% sucrose appears to be the minimum concentration required for optimum biofilm development using our *in vitro* model, which agrees well with previous studies [15, 20]. We evaluated next whether the combination of sucrose and various concentrations of starch enhance biofilm formation by *S. mutans*.

**Distinct sucrose and starch concentrations:** The biofilms were grown in 0.5% sucrose, 0.5% sucrose + 0.5% starch, 0.5% sucrose + 0.75% starch, 0.5% sucrose + 1% starch and 1% sucrose, and were analyzed as described above; the presence of starch alone results in negligible biofilms formation [15, 16]. The biomass and the amounts of insoluble polysaccharides increased with addition of increasing concentration of starch (from 0.5 to 1%) showing a positive correlation (R^2^ > 0.8; Table A2). *S. mutans* cells grown in the presence of 0.5% sucrose + 1% starch yielded the highest biomass, and presented more INS than other biofilms, which correlated well with *gtfB* expression (Fig. A1B). Clearly, the addition of starch enhanced (i) the ability of *S. mutans* to form biofilms in the presence of 0.5% sucrose, and (ii) the *gtfB* expression; which resulted in higher amounts of INS (vs. biofilms formed in 0.5 or 1% sucrose). These phenomena may be directly related to specific responses of *S. mutans* within biofilms to the presence of starch hydrolysates, undigested starch and sucrose at transcriptional level. Based on the preliminary data, we selected the 3 experimental groups (0.5% sucrose, 0.5% sucrose+1% starch and 1% sucrose) for further analyses of the transcriptome response by biofilms-cells of *S. mutans*.

**Determination of sugars released from starch digestion by surface-adsorbed salivary amylase.** Amylase-active sHA discs were incubated with 0.5% sucrose + 1% starch or 1% starch as described previously [15]. Starch is progressively digested by the surface-salivary amylase until the enzyme no longer catalyzes the hydrolysis, which is about 50% of initial starch concentration as determined experimentally [15]. At specific time points, samples were taken for carbohydrate analysis by size exclusion chromatography [52]. The samples were dissolved in 90% dimethylsulfoxide (DMSO) and then analyzed by a HPLC system equipped with a combination of Zorbax PSM columns and a Shimadzu 10A refractive index (RI) detector (Shimadzu Corporation, Kyoto, Japan). Each sample was analyzed in duplicate. The chromatographs showed two fractions, and the results are presented in Table A3.

**Table A1. Biomass (dry-weight), total amount of protein and extracellular polysaccharide (EPS) in *S. mutans* UA159 biofilms formed in the presence of varying** sucrose concentrations.

| **Experimental Groups** | **Dry-weight** | **Total amount of Protein** **(mg)** | **Total Amount of EPS** | |
| --- | --- | --- | --- | --- |
|  | **(mg)** |  | **WSP (mg)** | **INS (mg)** |
| 0.25% sucrose | 3.34 (0.34) A | 1.01 (0.08) A | 0.04 (0.01) A | 0.49 (0.10) A |
| 0.5% sucrose | 3.47 (0.83) AC | 0.97 (0.26) AB | 0.08 (0.05) AB | 0.78 (0.27) ABD |
| 0.75% sucrose | 5.01 (0.50) BC | 1.12 (0.13) A | 0.20 (0.10) BC | 1.18 (0.21) BC |
| 1% sucrose | 5.24 (0.61) B | 1.23 (0.32) A | 0.30 (0.09) CE | 1.29 (0.22) C |
| 2% sucrose | 5.29 (0.38) B | 1.26 (0.43) A | 0.51 (0.12) D | 1.20 (0.09) C |
| 5% sucrose | 4.33 (0.62) C | 0.73 (0.17) B | 0.55 (0.27) DE | 0.85 (0.13) D |

The values in parentheses are standard deviations. Values (n=4) in the same column followed by the different letters are significantly different from each other (P<0.05, ANOVA comparison for all pairs using Tukey’s test)

**Table A2. Biomass (dry-weight), total amount of protein and extracellular polysaccharide (EPS) in *S. mutans* UA159 biofilms formed in the presence of sucrose alone or in combination with** starch.

| **Experimental Groups** | **Dry-weight** | **Total amount of Protein** **(mg)** | **Total Amount of EPS** | |
| --- | --- | --- | --- | --- |
|  | **(mg)** |  | **WSP (mg)** | **INS (mg)** |
| 0.5% sucrose | 3.12 (0.23) A | 1.07 (0.07) A | 0.05 (0.01) A | 0.47 (0.06) A |
| 0.5% sucrose+0.5% starch | 3.81 (0.34) B | 1.16 (0.15) AB | 0.09 (0.02) B | 0.80 (0.21) B |
| 0.5% sucrose+0.75% starch | 4.50 (0.24) C | 1.13 (0.09) A | 0.20 (0.07) C | 1.02 (0.06) B |
| 0.5% sucrose+1% starch | 5.90 (1.05) D | 1.35 (0.14) B | 0.29 (0.09) C | 1.54 (0.29) C |
| 1% sucrose | 4.81 (0.37) CD | 1.31 (0.06) B | 0.21 (0.05) C | 1.01 (0.09) B |

The values in parentheses are standard deviations. Values (n=4) in the same column followed by the different letters are significantly different from each other (P<0.05, ANOVA comparison for all pairs using Tukey’s test)

**Table A3. The molecular weight and degree of polymerization (DP) distribution of starch hydrolysates digested by surface adsorbed α-amylase.**

| Incubation | Fraction 1 | | | | | |  | Fraction 2 | | | | | |
| --- | --- | --- | --- | --- | --- | --- | --- | --- | --- | --- | --- | --- | --- |
|  | Average MW (Da) | | Average DP | | Mass Ratio (%) | |  | Average MW (Da) | | Average DP | | Mass Ratio (%) | |
|  | Average | SD | Average | SD | Average | SD |  | Average | SD | Average | SD | Average | SD |
| 1 h | 2.15E+04 | 4784.5 | 132.5 | 29.5 | 71.6 | 1.1 |  | 4.90E+02 | 31.7 | 3.0 | 0.2 | 28.4 | 1.1 |
| 2 h | 1.44E+04 | 5258.1 | 88.6 | 32.5 | 58.3 | 1.4 |  | 4.75E+02 | 10.4 | 2.9 | 0.1 | 41.7 | 1.4 |
| 20 h | 1.45E+03 | 26.2 | 9.0 | 0.2 | 5.4 | 0.7 |  | 3.71E+02 | 7.8 | 2.3 | 0.0 | 94.6 | 0.7 |

Fraction 1 covers the elution volume between void volume and molecular size above DP4.

Fraction 2 covers the elution volume between DP1 (total volume) and DP4.

DP: degree of polymerization.


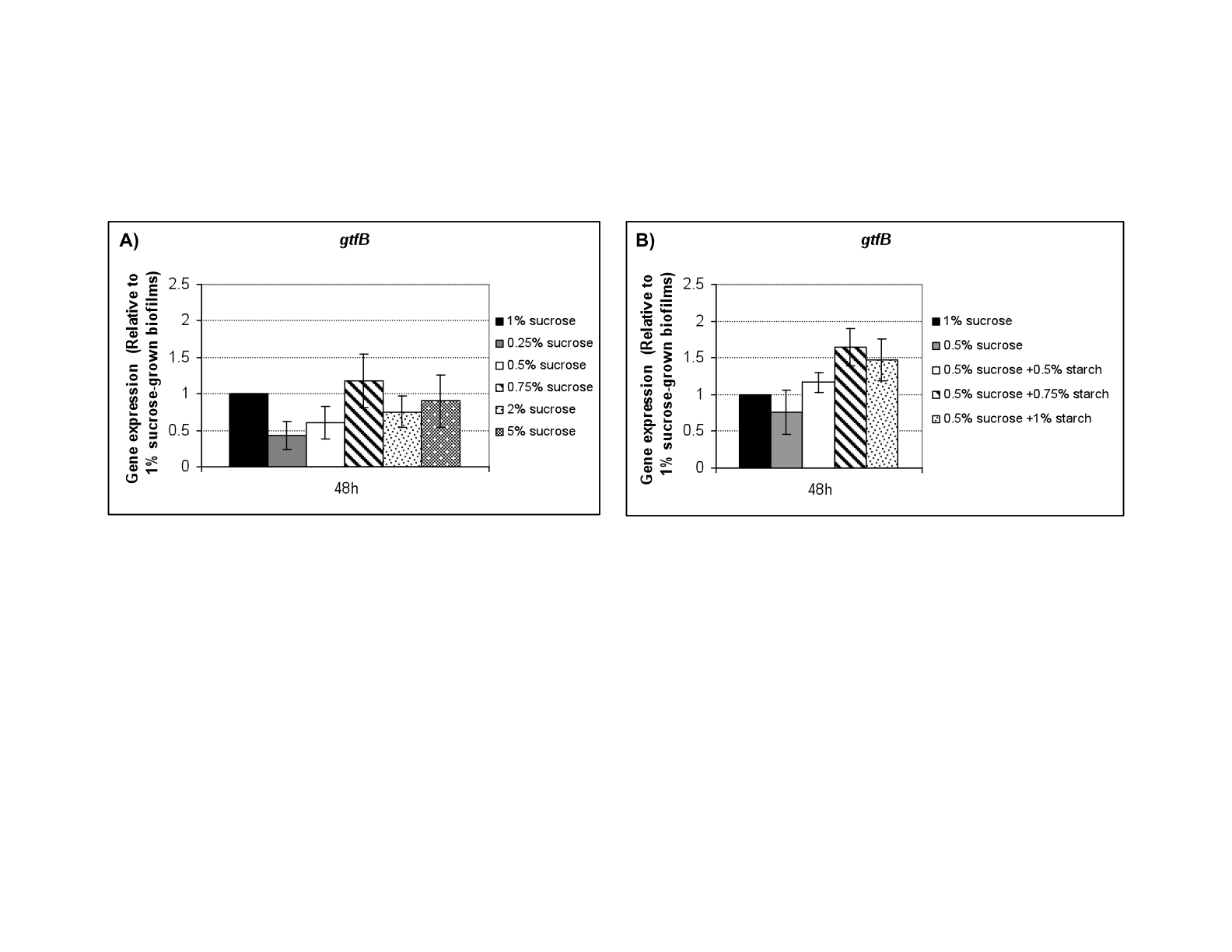


*

*

*

*

**Figure A1. RT-qPCR analysis of *gtfB* gene expression by *S. mutans* biofilms*,* grown in the presence of varying concentrations of sucrose (A), and sucrose + starch (B).** The mRNA level of *gtfB* in each sample was normalized to that of 16S rRNA. These values were then compared to those from 1% sucrose-grown biofilms (corresponding to an arbitrary value of 1) to determine the change (*n*-fold) in gene expression. Data are expressed as means ± standard deviations of triplicates from at least three separate experiments. An asterisk indicates that a value is significantly different from the value for the 1% sucrose-grown biofilms (P<0.05, Tukey’s test).
